# Peter Bent Brigham Hospital

**Published:** 1916-11-25

**Authors:** H. B. Howard

**Affiliations:** Superintendent. Boston


### THE EDITOR'S LETTER-BOX.

### Peter Bent Brigham Hospital.

*To the Editor of* The Hospital.

Sir,—In your first notice of this hospital in your issue of November 4, you speak of the toilets not having the "cut off." All our toilets are seat-vented, and this draft is made sure by having a fan connected with it. This same fan attends to the hoods in the duty-room and in the laboratory. The toilet-rooms are vented through the seat vents. No odour ever escapes into the room, much less into the corridor. It is a pretty universal custom in America to have the toilets open from the corridor. There is another nurses' toilet in each typical ward building in the basement, so that a nurse can always reach a toilet by going up or down one flight.

In the fourteen-bedded ward the framework is of iron, and the roof is of plank, plastered on the under side. This plain ironwork was left out in the open-air space of the ward at my request. The architects had intended to fur down that much, but I preferred to have the additional air space in the room, even if I had to have the iron framework there too.

We have a granolithic dado, five feet high, extending throughout the hospital. It is flush with the iron-door frames. This dado takes the hard knocks of the daily use. You might mention the absence of marble in our operating building. This dado takes the place of it. It is made of half Portland and Keene cement.

No mention is made of the beds being on wheels so that stretchers are not necessary, or the arrangement of the dining-rooms for all the help around one serving-room, or the sterilising hopper for typhoid stools, or the chair beds. So far as I am aware, we are the only hospital that has them. We have some thirty of them. I only mention these things so that if you wish to make your article longer you will have means at hand to do it. The rubber tyres on the wheels of our beds are made practically of canvas, with just enough rubber in to hold the canvas together, so that they never dent. We have used them now for three years. I am not aware that one of them has yet worn out.—Very sincerely yours,

H. B. Howard, Superintendent.

Boston, October 31, 1916.

